# Assessment of pathological features in Alzheimer’s disease brain tissue with a large field-of-view visible-light optical coherence microscope

**DOI:** 10.1117/1.NPh.5.3.035002

**Published:** 2018-07-24

**Authors:** Antonia Lichtenegger, Martina Muck, Pablo Eugui, Danielle J. Harper, Marco Augustin, Konrad Leskovar, Christoph K. Hitzenberger, Adelheid Woehrer, Bernhard Baumann

**Affiliations:** a Medical University of Vienna, Center for Medical Physics and Biomedical Engineering, Vienna, Austria; b General Hospital and Medical University of Vienna, Institute of Neurology, Vienna, Austria; c Vienna University of Technology, Institute of Applied Physics, Vienna, Austria

**Keywords:** optical coherence microscopy, Alzheimer’s disease, imaging system, visible light, supercontinuum laser

## Abstract

We implemented a wide field-of-view visible-light optical coherence microscope (OCM) for investigating *ex-vivo* brain tissue of patients diagnosed with Alzheimer’s disease (AD) and of a mouse model of AD. A submicrometer axial resolution in tissue was achieved using a broad visible light spectrum. The use of various objective lenses enabled reaching micrometer transversal resolution and the acquisition of images of microscopic brain features, such as cell structures, vessels, and white matter tracts. Amyloid-beta plaques in the range of 10 to 70  μm were visualized. Large field-of-view images of young and old mouse brain sections were imaged using an automated x−y−z stage. The plaque load was characterized, revealing an age-related increase. Human brain tissue affected by cerebral amyloid angiopathy was investigated and hyperscattering structures resembling amyloid beta accumulations in the vessel walls were identified. All results were in good agreement with histology. A comparison of plaque features in both human and mouse brain tissue was performed, revealing an increase in plaque load and a decrease in reflectivity for mouse as compared with human brain tissue. Based on the promising outcome of our experiments, visible light OCM might be a powerful tool for investigating microscopic features in *ex-vivo* brain tissue.

## 1 Introduction

One out of nine Americans aged above 65 years is suffering from Alzheimer’s disease (AD), making it the most common form of dementia worldwide.^1^ As our society is facing an aging population, the cases of AD will double in the next 20 years, leading to a considerable financial and social burden. With disease progression, patients are losing their ability to remember and in the end are dependent on care-giving.^1^ To diagnose AD while the patient is still alive, clinicians have to exclude other diseases with similar symptoms and check for cognitive and behavior changes with neurophysological tests. Ultimately, AD can only be diagnosed by a histological analysis of the brain tissue postmortem. However, as a step toward treatment of the disease, an early diagnosis is crucial.^2^

At the cellular level, the disease is characterized by the degeneration of neurons and the formation of neurofibrillary tangles composed of tau protein. Extracellular plaques composed of amyloid-beta (A-β) protein are also formed. In humans, the A-β plaques that are 10 to 200  μm in diameter are considered a hallmark of the disease and have been subject to many investigative studies.^3^^–^^5^ However, further research is needed to thoroughly understand the formation and accumulation of the A-β plaques in various brain regions and stages of the disease.^2^ For example, there are diverse opinions regarding the contribution of plaque formation to the etiology of the disease.^6^

Genetically modified animal models can help to gain a better understanding of the underlying mechanisms of the disease.^7^ Mouse models, in particular, have been extensively used as their breeding and handling is rather easy and cheap, and the genetic modification techniques are well established. In the past decades, a variety of mouse models have been developed, which reproduce different aspects of AD-related pathologies.^8^^,^^9^ The mouse model used in this paper exhibits an overexpression of the amyloid precursor protein (APP) and a presenilin 1 (PS1) mutation accompanied by a rapid development of A-β plaques in various cortical regions.^8^^,^^10^

Many imaging techniques have been used to investigate A-β plaques *in vivo* and *ex vivo* in both humans and animal models.^11^ In clinical research, computed tomography (CT) and magnetic resonance imaging (MRI) have been routinely used to explore potential biomarkers for the disease.^11^ In preclinical research, positron emission tomography (PET) and CT were used to study amyloid protein accumulation in whole brains of rodents;^12^^–^^14^ however, due to its high resolution in soft tissue, MRI has been the preferred tool for many studies.^15^^–^^17^ Resolutions as low as 35  μm were achieved using a mouse model with MRI.^17^ On the flipside, these neuroimaging techniques are rather expensive, time consuming, and complex.^17^^,^^18^ For much higher resolution, two-dimensional (2-D) and three-dimensional (3-D) images can be acquired using optical microscopy techniques, such as confocal microscopy or light sheet microscopy.^19^^–^^21^ In light sheet microscopy, optical clearing is frequently used to increase the penetration depth of light into the tissue to acquire 3-D images with micrometer-scale resolution. Using this method, large and densely sampled datasets are created; however, the acquisition time is often fairly long.^20^ A variety of techniques to perform optical clearing have been developed.^22^^,^^23^ In 2015, the clearing technique SWITCH was introduced, whose major advantages are the preservation of the tissue size, the possibility to label in multiple rounds, its simplicity, and cost efficiency.^24^

Optical coherence microscopy (OCM) was presented for investigations into A-β plaques at high resolution in 3-D.^25^^–^^28^ OCM enables investigations into tissue samples without any further staining using the intrinsic optical backscattering contrast. Thus, OCM can overcome tedious and time-consuming tissue preparations, which are usually required in traditional microscopy. OCM can also be used to perform spectroscopic imaging to investigate AD tissue samples.^28^ In 2012, A-β plaques were first identified by an OCM system.^25^ It is important to note that high numerical aperture objectives used in OCM limit the Rayleigh range of the system. To overcome this tradeoff, a Bessel beam illumination in the near-infrared wavelength region around 800 nm was utilized.^25^ Polarization-sensitive systems are used to gain additional contrast in OCM images.^26^^,^^29^ A polarization-sensitive OCM system operating at 840 nm was utilized to identify plaques based on their intrinsic birefringence properties.^26^ To achieve higher axial resolutions in OCT, broader spectral coverage with shorter central wavelengths is needed.^27^^,^^28^^,^^30^^–^^35^ Recently, using broad visible spectrum OCT systems, submicrometer axial resolutions were accomplished and neuritic A-β plaques with diameters in the micrometer range were identified.^27^^,^^28^

In this paper, we present visible light OCM for investigating microscopic features of brain tissue samples from human AD patients and from an AD mouse model. By implementing different objective lenses, we investigated the impact of different magnifications on the imaging performance for various features of the brain such as cellular and vascular structures as well as white matter tracts. A-β plaques in the range of 10 to 70  μm were identified as highly scattering features in the brain tissue. *Ex-vivo* brain tissue affected by cerebral amyloid angiopathy (CAA) was investigated and A-β accumulations were imaged in vessel walls. Additionally, by implementing an automatic x−y−z stage, large area scans were acquired. A first effort was made to quantify the plaque load in sections of young and old AD mouse brains. Finally, a comparison of A-β plaques observed by OCM in human brains and their counterparts in mouse brain tissue was performed.

## 2 Methods

### 2.1 Visible-Light Optical Coherence Microscope

**Fig. 1:**
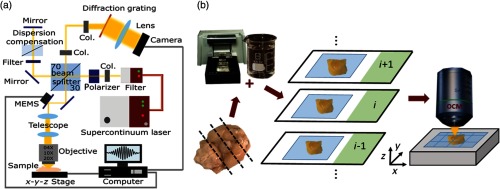
The visible-light OCM and the tissue processing pipeline. (a) The visible light OCM system (Col. = Collimator). (b) The extracted brain tissue is embedded in 5% agarose. A vibratome is used to section the brain-agarose block in 200-μm slices. These sections are glued onto glass slides and are then optically cleared for 15 min following the SWITCH protocol.^24^ The cleared sections are imaged with the OCM setup. An automated x−y−z stage is integrated in the sample arm to acquire images with a large field-of-view.

The visible light OCM system used in this work is described in detail elsewhere^28^ and thus is only explained briefly in the following. The sketch of the OCM setup is shown in Fig. 1(a). The OCM system comprises a free-space Michelson interferometer and a homemade spectrometer acquiring spectral data at an A-scan rate of 30 kHz. A supercontinuum laser (NKT Photonics SuperK EXTREME EXU-6) in combination with a tunable filter box (NKT Photonics SuperK VARIA) delivers the visible light spectrum, centered at 555 nm with a full width at half maximum of 156 nm. The resulting axial resolution was measured using a mirror as a sample to be 1.2  μm in air, which corresponds to 0.88  μm in brain tissue assuming a group refractive index of 1.36.^36^ The original system^28^ was modified in several ways. Objectives with 4× (UPLFLN 4XP, Olympus), 10× (UPLFLN 10XP, Olympus), and 20× (UPLFLN 20XP, Olympus) magnification were used to acquire images with different fields of view and thus with different transversal resolutions. Transversal resolutions from 8 to 2  μm were measured using the US Air Force 1951 resolution test target and the field-of-view varied from $1000\times 1000\;\mu m^{2}$ down to $300\times 300\;\mu m^{2}$, respectively. The theoretical depth of focus values were calculated to be 711, 177, and 44  μm, respectively. While imaging the focus was set at the tissue surface. For each objective, the length of the reference arm and the focus had to be adjusted, and dispersion compensation was optimized both in hardware and in postprocessing.

Imaging was also performed with a 10× water immersion objective (UMPLFLN 10XW, Olympus) to reduce reflection artifacts from the superior surface of the tissue. Additionally, two motorized stages (MLS203-1 and MZS500-E, Thorlabs) moving in x-, y-, and z-directions, respectively, were integrated into the sample arm and automated for scanning large fields-of-view up to several square millimeters. The maximum scanned area was $2.5\times 2.5\;{\mathrm{mm}}^{2}$. A custom-made LabView (LabView 2015, Version 15.0, 64-bit, National Instruments) program controlled the automatic x−y stage motion and the focus was set with the z-stage. Furthermore, a water-filled glass cuvette was integrated into the reference arm for improved balancing of dispersion mismatch introduced by the tissue and the water between the tissue and the immersion objective. A sensitivity of 91 dB with a sensitivity roll-off of 24  dB/mm was measured where the shot noise limit was calculated to be 94 dB.^37^

### 2.2 Tissue Processing Pipeline

A mouse tissue processing pipeline was developed, which is shown in Fig. 1(b). After carefully extracting the brain, the tissue was fixed in 4% paraformaldehyde for seven days. The fixed brain tissue was embedded in a 5% agarose gel. This block was sectioned into 200-μm thick slices using a vibratome (Vibratome Series 1000 Sectioning System, The Vibratome Company). The sections were then glued onto glass slides. Then optical clearing was performed for 15 min at 37°C following the SWITCH protocol.^24^ In the previous works, the advantages of combining OCM with optical clearing were explored.^28^^,^^38^ The duration of 15 min used here was found to be optimal for optical clearing to reach deeper light penetration into the tissue while keeping enough contrast for OCM imaging.^28^ All results were compared with histological images. One hemisphere of the brain was processed following the steps explained above while the other was used for histology. Results from OCM imaging were then compared with histological images gained at a similar position in the contralateral hemisphere. For these images, the hemisphere was embedded in paraffin and sectioned into 3-μm coronal slices using a microtome. Immunohistochemistry against A-β was performed on every third section [Dako Beta-Amyloid 1:50 (M0872, Clone 6F/3D), Detection system Dako EnVision]. Some of the remaining sections were stained with Congo red to confirm A-β plaque findings. In all images, hematoxylin was used as a nuclear staining. Micrographs were acquired with a slide scanner (C9600-12, Hamamatsu) and after acquisition the plaques were segmented automatically in the micrographs using the “ColSeg” plugin of Fiji.^39^^,^^40^ To analyze the plaques in AD-affected human brain tissue, a small piece was excised from the cortex, embedded in agarose gel, and cleared following the same procedure as described above. The surface was imaged using OCM and afterward histology was performed to confirm the results. For imaging the CAA-affected arteries, no clearing step was conducted.

#### 2.2.1 APP-PS1 mouse brain samples

Heterozygous breeding of APPswe, PSEN1dE9 (APP-PS1, MMRRC stock number 34829, The Jackson Laboratory^10^^,^^41^) mice was established. Brains of both the APP-PS1 mouse model and their healthy litter mates were investigated. Animal experiments were approved by the local ethics committee and by the Austrian Federal Ministry of Science, Research and Economy under protocol BMWFW-66.009/0360-WF/V/3b/2016. Mice with an age of 8 (N=2), 51 (N=2), and 64 (N=2) weeks were sacrificed and investigated.

#### 2.2.2 Human brain samples

The human brain samples were provided by the Neurobiobank of the Medical University of Vienna (ethics approval number 396-2011). Specimens were obtained from patients, who underwent autopsy at the Medical University of Vienna. Formalin-fixed *ex-vivo* brains of one control patient and two late stage AD patients were investigated primarily in the frontal cortex. In both pathological cases, CAA was previously confirmed by histology.

### 2.3 Data Acquisition and Postprocessing

An imaging protocol consisting of 8192×500 (A-scans)×500 (B-scans) pixels per volume was chosen. The raw data were acquired using a custom-made LabView program. The postprocessing pipeline was implemented in MATLAB (MATLAB, R2015b, MathWorks). An additional step of spectral shaping was integrated to reduce side lobe effects in the images after resampling linearly the data to k-space.^42^ Attenuation images were calculated as described in previous works.^28^^,^^43^ For the OCM *en-face* projections, the intensity values over the first 50  μm underneath the tissue surface were averaged unless otherwise stated.

#### 2.3.1 Plaque load evaluation in large field-of-view images

For the large field-of-view images, the plaque load (plaques per square millimeter) was evaluated. In the OCM intensity *en-face* projections, the plaques were manually segmented. To have a fair comparison to histology, five consecutive histological slices with two slices in between (accounting for the 50  μm averaged in OCM) were chosen in a similar brain region in the corresponding contralateral hemisphere. The histological images were registered using the Fiji “StackReg” tool,^44^ and the plaques were segmented using color deconvolution and thresholding in Fiji. The union of these resulting five binary images was automatically analyzed using Fiji. The plaque diameter and the total plaque number were extracted.

#### 2.3.2 Comparing plaques in human and mouse brain tissue

The plaques in the OCM intensity volumes were segmented manually using the segmentation paint brush tool in ITK-Snap.^45^ The plaque load (plaques per cubic millimeter), the plaque volume (${\mu m}^{3}$), and the intensity values in the OCM images were evaluated using Fiji^39^ and MATLAB. For the intensity analysis, 25 randomly selected plaques in each volume for human and mouse brain tissue were analyzed by its contrast-to-noise ratio (CNR) that was calculated using^46^

$$\mathrm{CNR}=\frac{\left|\mu_{P}-\mu_{B}\right|}{\sqrt{\sigma_{P}^{2}+\sigma_{B}^{2}}}.$$ (1)

Using the mean intensity values of each plaque ($\mu_{P}$), the mean intensity in the surrounding brain parenchyma ($\mu_{B}$), and the variance $\left(\sigma_{P}^{2},\sigma_{B}^{2}\right)$ of these intensity values. The surrounding background in the brain tissue was chosen at the same depth as the plaques.

### 2.4 Statistics

To compare the extracted brain features, box plots and histograms were generated for a descriptive analysis. Two-sample Kolmogorov–Smirnov tests with Bonferroni correction were performed in MATLAB for statistical analysis, with a significance level of p<0.05.

## 3 Results

### 3.1 Investigating Microscopic Features of Mouse Brain Tissue

**Fig. 2:**
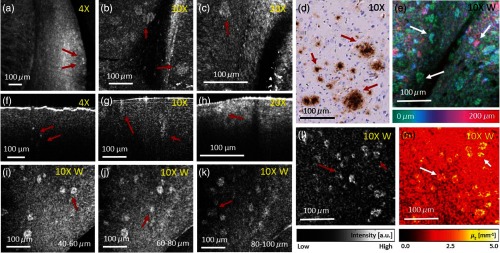
Amyloid-beta (A-β) plaques were investigated using visible light OCM at three different magnifications. (a)–(c) OCM *en-face* projection images taken with 4×, 10×, and 20× magnification objectives, respectively in a 51-week-old mouse. (d) Immunohistochemically and hematoxylin stained tissue section of a plaque-rich region. Plaques appear brown. (e) Depth intensity projection over 200  μm in a plaque-rich region in a 51-weeks-old mouse brain cortex. (f)–(h) Representative OCM B-scans taken with 4×, 10×, and 20× magnification objectives, respectively. (i)–(k) OCM *en-face* projections over 20  μm for different depth positions (40 to 100  μm) of a 64-weeks-old mouse brain. Plaques appear at different depths in the brain tissue. (l) OCM *en-face* projection image taken with the 10× magnification water immersion objective of a 64-weeks-old mouse brain. (m) Attenuation image of (l). For all *en-face* projections, the intensity over the first 50  μm underneath the tissue surface were averaged. In all images, representative A-β plaques are marked with red and white arrows.

Using different objectives with 4×, 10×, and 20× magnifications, imaging of mouse brain tissue was conducted and plaques, cellular structures, white matter tracts as well as vascular features were visualized. APP-PS1 mouse (8 to 64 weeks old) brain sections were imaged using the three magnification levels and A-β plaques ranging from 10 to 70  μm in diameter were observed in the cortex. Figures 2(a)–2(c) show OCM *en-face* projections of different regions at different scales. In all images, examples of A-β plaques are marked with red arrows. An immunohistochemically stained image of a neighboring region from the same mouse brain is shown in Fig. 2(d). Representative B-scans at the three magnifications including plaques are shown in Figs. 2(f)–2(h). An *en-face* projection using a water immersion 10× objective is shown in Fig. 2(l). Imaging with the water immersion objective improves visibility of smaller plaques that exhibit less contrast [Fig. 2(l)] as compared with the image taken without water immersion [Fig. 2(b)].

By optical clearing brain tissue, the penetration depth of light was doubled which makes it possible to image thicker volumes with a single acquisition.^28^ One advantage when imaging AD-affected brain tissue with OCM is the ability to visualize plaques quickly in 3-D. Figures 1(i)–1(k) show OCM *en-face* projections over 20  μm in the tissue at different depth positions acquired with the 10× water immersion objective. In these projections, plaques can be observed throughout the whole OCM volume. In Fig. 2(e), a color-encoded depth projection shows the appearance of plaques in another dataset in a region over 200  μm. An attenuation image [Fig. 2(m)] calculated from Fig. 2(l) shows that the attenuation in plaques is higher compared with the surrounding tissue.

**Fig. 3:**
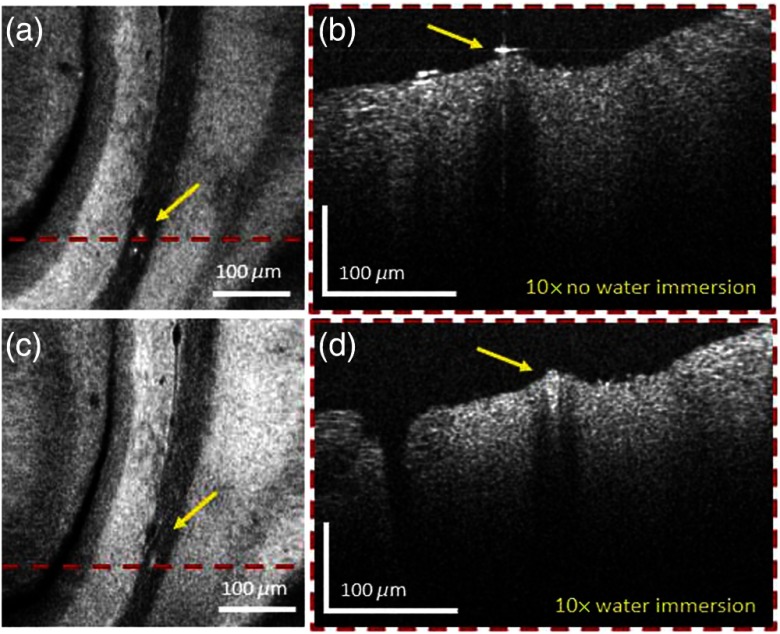
Investigating AD mouse brain tissue with and without water immersion. (a) *En-face* projections without water immersion and (b) with water immersion objective using 10× magnification. (b) and (d) The corresponding B-scan images. Images (a)–(d) were acquired in a 51-weeks-old mouse brain. The yellow arrows indicate the positions where artifacts are introduced by the reflection from the tissue surface.

To highlight the advantages of imaging with a water immersion objective, the same position in the mouse brain was first imaged using the 10× magnification objective without water and then with water immersion. The results are shown in Figs. 3(a)–3(d). Especially in the B-scan image without water immersion, Fig. 3(b) reflections from the first surface are clearly visible, which are suppressed in Fig. 3(d). Details are obscured by the reflections [this is marked by yellow arrows in Figs. 3(b) and 3(d)]. Also in the *en-face* projection Fig. 3(a) artifacts appear.

**Fig. 4:**
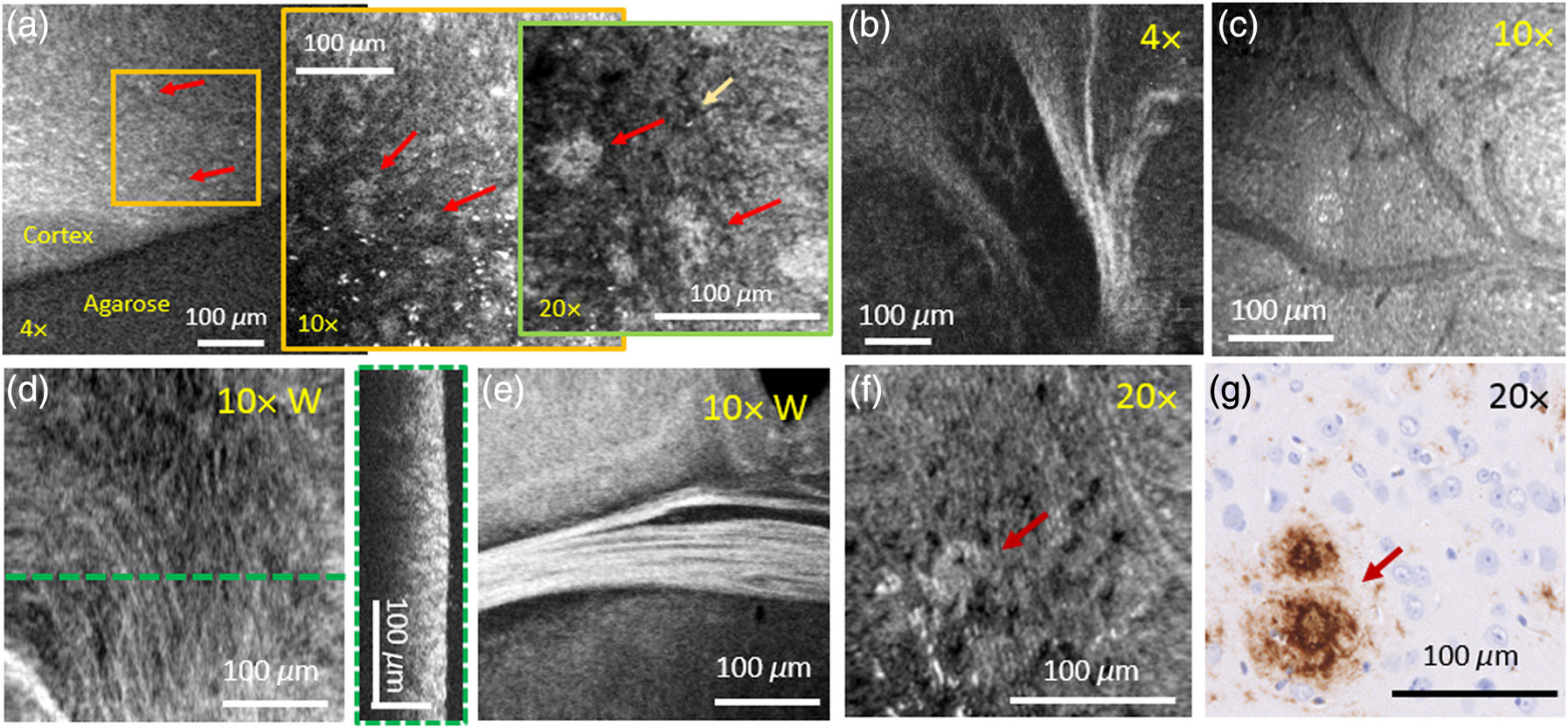
Microscopic features of AD and control mouse brain tissue imaged with various magnifications. (a) *En-face* projections of the same mouse cortex position imaged first with a 4× then with a 10× and last with a 20× objective in a 64-weeks-old mouse brain. (b)–(f) White matter tracts (b), (d)–(e) vascular features (c), and single cellular structures (f) are visible under various magnifications in *en-face* projections. (c) *En-face* OCM projection image of a vascular region. (d) *En-face* projection and the corresponding B-scan OCM image. (e) OCM intensity images taken with a 10× magnification water immersion objective. White matter tracts appear as highly scattering features in the *en-face* OCM projection image. (g) Histological image showing a similar plaque region as imaged with the 20× magnification objective in (f). For all *en-face* projections, the intensity values over the first 50  μm underneath the tissue surface were averaged. In all images, representative A-β plaques are marked with red arrows. Images (b)–(d) were acquired in a 51-week-old mouse brain and for the other images a 64-week-old mouse brain was used.

Control and APP-PS1 mouse brain sections were imaged to investigate the advantages of using various objectives. The same position in a mouse brain cortex was imaged with 4×, 10×, and 20× magnification. The results are shown in Fig. 4(a). Using the 4× magnification, an overview of the imaged region is achieved. Going to 10× magnification, amyloid-beta plaques can clearly be identified for further analysis, for example, for plaque load evaluation as it was done in this article. Using 20× magnification, the structure of the plaques can be investigated in greater detail; however, the field of view is very limited.

Further microscopic features were investigated using the three magnification levels. Figure 4(b) shows white matter tracts imaged with the 4× magnification objective. In Fig. 4(c), the vasculature of a brain region imaged by a 10× magnification objective can be seen. Figures 4(d)–4(e) show OCM images taken with the 10× water immersion objective. Figures 4(d) and 4(e) show *en-face* projections over the first 50  μm underneath the tissue surface where white matter tracts are clearly visible. In Fig. 4(d), even smaller fiber tracts can be identified, which are also be observed in the B-scan image as regions with higher intensity. When investigating brain tissue with 20× magnification, see Fig. 4(f), A-β plaques can be resolved at higher transversal resolution and cellular structures can be observed as hyposcattering spots. For comparison, Fig. 4(g) shows a histological image of an adjacent region in the same mouse brain.

### 3.2 Large Field-of-View Images of Mouse Brain Tissue

**Fig. 5:**
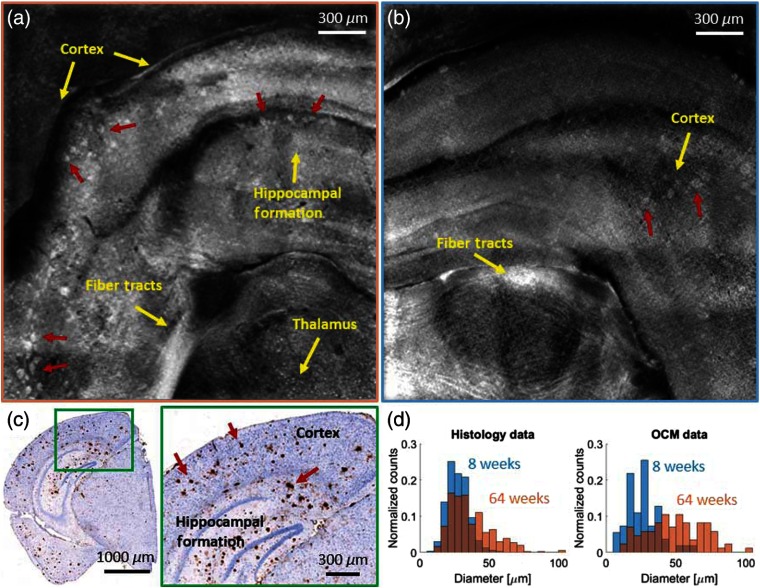
Large field-of-view images of brain tissue of an (a) 64-week- and a (b) 8-week-old AD mouse, acquired with the 10× water immersion objective. (c) Immunohistochemically and hematoxylin stained histological section of the AD mouse shown in (a). (d) Histograms showing the plaque diameter distribution in the 8- and the 64-weeks-old mice in histology and OCM images. Amyloid-beta plaques in all panels are indicated by red arrows and anatomical features such as white matter tracts are indicated in yellow.

Large field-of-view images of brain sections from two young (8 weeks) and two old (64 weeks) AD model mice were acquired. A coronal section in the cortex and the hippocampal region was imaged. A first analysis of the plaque load was performed as a proof of concept. In Fig. 5(a), a large field-of-view *en-face* image of a brain section from a 64-week-old AD mouse acquired with the 10× water immersion objective is shown. In total, 49 tiles were automatically acquired and manually stitched together to cover an approximate area of $2.5\times 2.5\;{\mathrm{mm}}^{2}$. An immunohistochemically stained section of the same brain region is shown in Fig. 5(c), with a zoom-in into the cortex and the hippocampal formation. In both the OCM intensity images and the histologic section, anatomical features such as the cortex, the hippocampal formation, and the thalamus can be identified.^47^ At the bottom of the large field-of-view images, large white matter fiber tracts can also be observed. A large number of amyloid-beta plaques can be identified in the OCM intensity images as hyperscattering structures [Fig. 5(a), marked with red arrows] as well as in the histologic sections as dark brown spots [Fig. 5(c) marked with red arrows]. In Fig. 5(b), a similar brain region was imaged in an adolescent mouse (8 weeks) using a total of 36 tiles. Less overlap was chosen to cover the same area as compared with the scan of the old mouse brain section to reduce the acquisition and processing time. Two additional brain sections (one 8-week old and one 64-week old) were imaged using 36 tiles. To have a quantitative comparison of the plaque load, the plaques were manually counted in the OCM intensity images as well as automatically counted in the histology images using Fiji,^39^ and the mean values were compared for all four brains. As the *en-face* OCM data were averaged over 50  μm in the tissue; for the histological evaluation, a stack of slices was averaged to cover the same depth. In comparison with the image mosaic of the old mouse, less plaques are visible and their contrast to the surrounding tissue is rather low. In the older mice, an average of $23\;\text{plaques}/{\mathrm{mm}}^{2}$ were counted in the OCM image mosaic and $24\;\text{plaques}/{\mathrm{mm}}^{2}$ in the histology image. The imaged positions were acquired in similar regions and the results from OCM imaging and histology agree well. For the young mice, an average of $17\;\text{plaques}/{\mathrm{mm}}^{2}$ were counted in the OCM image, compared with $16\;\text{plaques}/{\mathrm{mm}}^{2}$ in the histology image. In total, this results in an increase of 35% for the plaque load in OCM and 50% in histology in aged compared with young mouse brains. These values are in good agreement with numbers found in the literature for APP-PS1 mice of similar age.^20^^,^^48^^,^^49^ In both histology and OCM images, the plaques appear smaller in younger mice. For quantification histograms are shown analyzing the plaque diameter in the 8- and the 64-week-old mouse brains. The trend of bigger plaques in the older mice can be shown in Fig. 5(d) and significant differences were found in OCM and histology analysis (p<0.01). From the histogram, it seems that plaques imaged with OCM in the 64 weeks old mouse appear to be bigger compared with histology. The statistical analysis resulted in a significant difference between plaque size in the 64-week-old mice measured with OCM compared with histology (p<0.01); however, for the 8-week-old mice no difference was found (p=0.07).

### 3.3 Comparing Plaques in Human and Mouse Brain Tissue

**Fig. 6:**
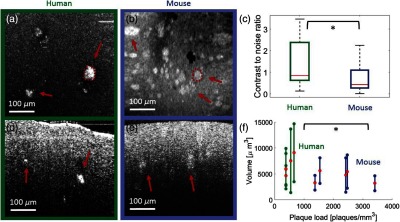
Comparison of amyloid-beta (A-β) plaques detected by OCM in human and mouse frontal cortex tissue imaged with a 10× magnification objective. *En-face* projection image in (a) human brain tissue and in (b) mouse brain tissue. In panels (a) and (b), examples of manually segmented plaques are included. (c) Boxplot showing the difference in contrast-to-noise ratio (CNR) measured by OCM within the segmented plaques (N=50). (d) Representative B-scan from AD-affected human brain tissue. (e) Representative B-scan of brain tissue from the AD mouse model. (f) Statistical analysis of the plaque volume over the plaque load in human and mouse brain tissue. The mean values are marked with red dots and the respective standard deviations are indicated by the whiskers for the volume data. The asterisk indicates the significance level p<0.05. For both *en-face* projections, the intensity values over the first 50  μm underneath the tissue surface were averaged.

A-β plaques in human brains and in brains of the AD mouse model were manually segmented. Based on the size, number, and CNR values gained from 10 datasets (five data sets from two mice and five data sets from two human patients imaged with a 10× magnification objective), differences were characterized. All data were acquired in the frontal cortex. The brain tissue was classified as late stage AD and was compared with brain tissue taken from aged mice (64 weeks). Figures 6(a) and 6(b) show *en-face* projection images in human and mouse brain tissue, respectively. Two representative plaque segmentations are outlined with red lines. In Fig. 6(c), a boxplot shows significantly higher CNR values measured in human plaques compared with plaques found in mouse brain tissue (p=0.04). Each data point represents one OCM volume of a brain slice. Figures 6(d) and 6(e) show representative B-scans of plaque regions in human and mouse brain tissue, respectively. Figure 6(f) shows that the plaque load [plaques per cubic millimeter ($\text{plaques}/{\mathrm{mm}}^{3}$)] in mouse tissue is significantly higher (p=0.03) compared with human tissue. Human plaques tend to be bigger (human $6600\pm 1700\;{\mu m}^{3}$, mouse $4500+1200\;{\mu m}^{3}$), but no significance was found (p=0.057). Each pair of whiskers represents one dataset, where the length of the whiskers indicates the respective standard deviations for each volume.

### 3.4 Imaging Cerebral Amyloid Angiopathy-Affected Arteries of Human Alzheimer’s Disease Patients

**Fig. 7:**
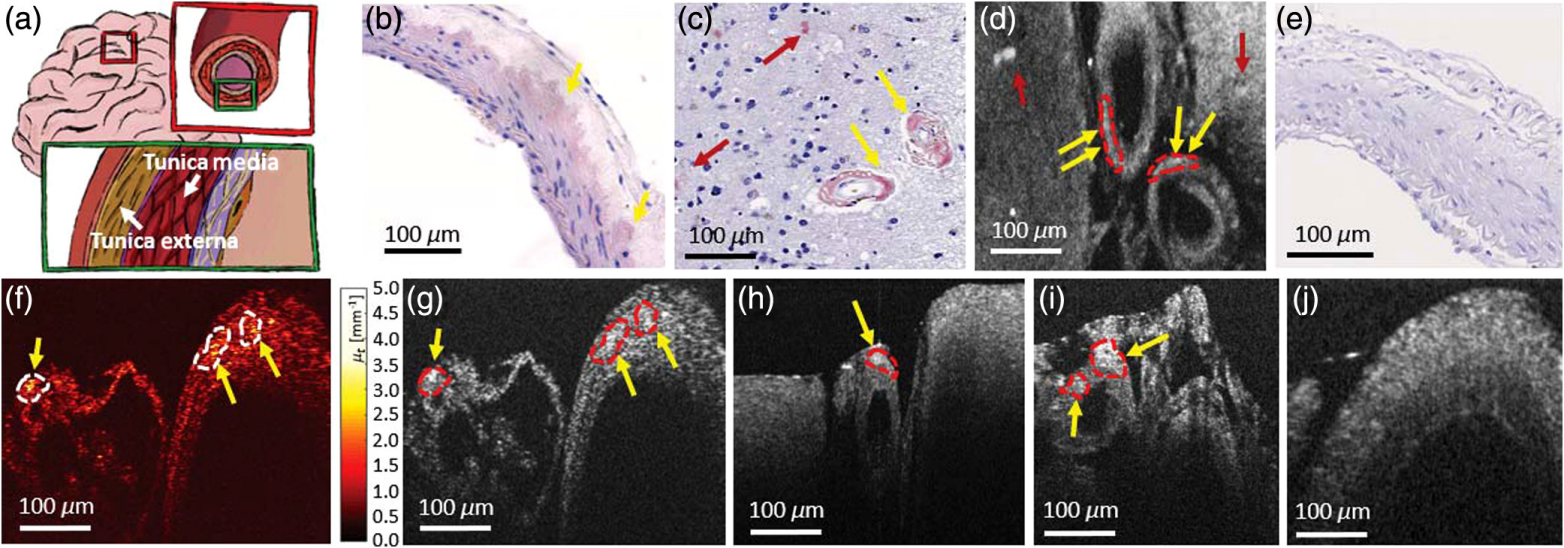
OCM of CAA. (a) Schematic drawing of a cerebral artery with the arterial wall structure. White arrows indicate the most frequent positions where A-β accumulates. (b)–(c) Histological images (Congo red and hematoxylin staining). A-β accumulation in the arterial wall structure and A-β plaques can be seen. (d) *En-face* projection over the first 50  μm underneath the tissue surface. (e) Histological image (Congo red and hematoxylin staining) of a control cerebral artery, where no A-β accumulations can be found. (f) Attenuation B-scan image. (g) Corresponding intensity B-scan of (f). (h)–(i) Intensity B-scans acquired in CAA-affected brains. (j) OCM B-scan image of the control cerebral artery. Yellow arrows indicate the A-β accumulation in the arteries and red arrows A-β plaques in the surrounding tissue. Examples of manually segmented accumulations are included as well.

CAA is characterized by the deposition of A-β in small and medium-sized arteries, capillaries, and less frequently in the veins of the cerebral cortex.^50^ For imaging, a 10× magnification objective was utilized. Figure 7(a) shows a schematic drawing of a vessel wall. A-β mostly accumulates in the tunica media and tunica externa (white arrows).^51^ Approximately, one-quarter of brains diagnosed with AD are also diagnosed with CAA.^50^ In histologic sections of the investigated CAA brains, A-β accumulations were found, see Figs. 7(b) and 7(c).

In Fig. 7(d), an *en-face* projection shows the A-β accumulations in the vessel wall. Such accumulations are marked with yellow arrows, whereas A-β plaques in the surrounding brain tissue are marked with red arrows. Figure 7(e) shows the histological section of a control brain where no A-β accumulations can be found. All OCM images were taken with the 10× magnification water immersion objective. In Fig. 7(f), the A-β accumulations in the arterial wall can be clearly identified through higher attenuation values. Figure 7(g) shows the corresponding intensity B-scan. In the OCM images, the vessels can be identified by their specific wall structure known from histology. In the representative B-scans in Figs. 7(h)–7(i), A-β accumulations in the arterial walls and A-β plaques can be seen as highly scattering features. Figure 7(j) shows an OCM B-scan image of a control brain in which no A-β accumulations were found in the arterial wall.

## 4 Discussion

In this article, a visible light OCM system was utilized to investigate brain tissue of human AD and CAA patients and of a mouse model of AD. The high axial resolution of this OCM prototype resulted from the broad spectral bandwidth (255 nm) of the light source, centered in the visible wavelength range (425 to 680 nm). The OCM system enabled the imaging of anatomical features with submicrometer resolution up to 200-μm deep in tissue. Through the ability of the system to use a variety of objectives and an automated x−y−z sample stage, wide field-of-view images with different magnifications were acquired. Using water immersion objective reflections from the supreme tissue surface can be oppressed. This leads to a better image contrast and additional details are revealed, as it is shown in Figs. 3(a)–3(d). A-β plaques, A-β accumulations in vessel walls, vascular features, cellular structures, and white matter fiber tracts were investigated. Unlike the surrounding brain tissue, white matter fiber tracts are highly scattering structures and therefore appear bright in the OCM intensity images. The same behavior has been previously described by other groups.^27^^,^^38^^,^^52^^,^^53^ The different magnifications enabled the visualization of white matter tracts in different scales, see Fig. 4. Cellular structures were successfully imaged with the visible light OCM system, appearing as hyposcattering structures in the image [Fig. 4(g)]. This is also in agreement with previous reports, although there are some controversial discussions if the investigated structures are cell nuclei or cell bodies.^27^^,^^35^^,^^52^^,^^54^^,^^55^ The big advantage of using various magnifications is that the tissue can be investigated at different scales. An overview is provided when imaging with the 4× objective. Following this plaque analysis is possible when imaging with the 10× objective. Finally, the 20× magnification allows high detail imaging at the cellular level.

A tissue processing pipeline was established to provide a fast and reproducible approach for brain tissue imaging using a visible light OCM system. Working with visible light, the penetration depth is limited compared with near infrared light; therefore, a tissue optical clearing step was integrated to extend the penetration. Using the SWITCH protocol, the penetration depth was doubled for assessing thick sections. This enables the 3-D morphological investigation of amyloid-beta plaques in deeper tissue layers, as it was shown in Fig. 2(e). The advantages of this clearing method are that it is rather easy and cheap to implement and that it does not induce tissue shrinkage. One drawback of SWITCH is that multiple tissue processing steps have to be performed which is time consuming. Hence, it might be interesting to investigate other clearing protocols, such as ethyl cinnamate (ECi), which involve less steps.^56^

As an extension to the system, an automatic motorized x−y−z stage was integrated in the sample arm to enable acquisitions of large field-of-view images over several square millimeters. In principle, with the custom-made software and the stage, fields-of-view into the square centimeter range could be acquired. The stage can be used to acquire image ranges up to 11.0  cm×7.5  cm.

The OCM system was utilized to acquire images in brain sections of young and old AD mice. Age-related plaque load increase was evaluated by assessing the number of plaques per cubic millimeter. The neuritic plaques were counted both in OCM images and corresponding histologic sections. As the results of both approaches agreed well, visible light OCM may be considered as a suitable tool for analyzing the plaque load. A 35% to 50% increase of plaque load was observed between 8-weeks-old and 64-weeks-old mice, which is in good agreement with values reported in the literature.^20^^,^^48^^,^^49^ From histology and OCM images, it was observed that the plaques seemed to be smaller and fewer in younger mouse brains compared with the older ones. A histogram analysis showed that in the 64-week-old mice significantly bigger plaques appear (p<0.01). The histogram analysis also showed that plaques appeared bigger when imaged with OCM and had a broader distribution of the diameter when compared with micrographs of histological sections. For the 64-week-old AD mouse, this diameter difference was statistically significant (p=0.01), however this was not the case for the 8-week-old mice (p=0.29). The difference may be explained by the different contrast mechanisms underlying the two modalities. Immunohistochemical staining specifically marks amyloid-beta such that it is possible to precisely segment the plaques in the histology images. In OCM imaging, the contrast is based on the scattering differences between structures. As the density of amyloid fibrils is not constant within the plaques,^57^ this may lead to a fuzzier border between plaques and the surrounding brain parenchyma, resulting in a broader distribution in diameter and a bigger size of the segmented plaques. In the literature, a similar topographic distribution of amyloid-beta plaques in human and mouse brains was observed; however, at a different time scale as the life cycles are very different.^20^^,^^58^ For the future, it would be interesting to further characterize an optimal age of the mouse model that realistically represents the plaque accumulation of AD patients. Based on our promising observations, we plan to thoroughly analyze brain sections of AD mice at different ages in a cross-sectional study to further characterize the mouse model. Also, until now, the stitching of the OCM images was performed manually and only for *en-face* projections, but mosaicing of OCM volumes may be fully automated by advanced processing routines in the future. One problem that has to be overcome is to automate the z-stage adjustment to set the focus. A software will be implemented, which will detect the first surface of the tissue and then set the focus accordingly.

A-β plaques in the range of 10 to 70  μm in diameter were visualized as they appear as strongly scattering structures compared with the surrounding cortical tissue. The size range and the localization of the plaques mainly in the cortex and the hippocampal formation are in agreement with values reported in the literature.^8^^,^^20^^,^^49^

In the attenuation images [Figs. 2(m) and 7(f)], smaller plaques may often be visualized more clearly than in the corresponding OCM intensity images [Figs. 2(l) and 7(g)]. Hence, attenuation analysis may be a useful tool toward an automated segmentation of plaques in brain tissue. Furthermore, the results of our OCM measurements were in good agreement with observations in histologic sections of the same sample. A-β plaques can be categorized into neuritic and diffuse plaques.^6^ Staging of the plaques based on the OCM image contrast could be investigated in the future. The chemical composition of the plaques and probably the resulting different refractive index could be an explanation for the contrast in the intensity images.^59^ Another source of the contrast of the plaques in the OCM intensity images could be the specific backscattering profile of the plaques, which was described in a mathematical model by Eugui et al.^60^ As mentioned in the methods, when changing between objectives, the dispersion mismatch had to be corrected in postprocessing. This could be overcome using the same objectives in both the sample and reference arm or by having two reference arms and switching between them, as it was shown by Lefebvre et al.^61^

A comparison of the CNR, size, and load between human and mouse brain tissue was performed. Our results suggest that mouse brain tissue exhibits a higher plaque load when compared with human brain tissue in the cortex. For the plaque size, the human brains tend to have bigger plaques (human $6600\pm 1700\;{\mu m}^{3}$, mouse $4500\pm 1200\;{\mu m}^{3}$) but no significant difference was found by OCM. However, until now only two patients and two mice were imaged and in the future more datasets will be acquired and evaluated. Still, similar results were found in the literature.^49^^,^^59^ The CNR in human plaques was significantly higher compared with murine plaques. Literature suggests that the chemical composition of human and mouse plaques is different.^59^ In human brains, more dense plaques are developed compared with those in the brains of AD mouse models, which might affect the light scattering properties and thus could be an explanation for the higher CNR values observed in the OCM intensity images.^59^^,^^62^ Another reason for the observed higher intensity values in human tissue could also be the longer fixation time of the tissue.^63^ One approach could be to compare fresh samples of human and mouse brain tissue. For our analysis, plaques in the cortex region were imaged, in the future the A-β plaques in other brain regions will be analyzed to track temporal changes in the plaque distribution.

CAA is a common pathology diagnosed in AD patients and is characterized by A-β accumulation in the arterial walls. CAA-affected *ex vivo* brain tissue of human patients was investigated and hyperscattering regions were observed in the arterial wall regions. The results were compared with histology showing good agreement.

The next upgrade to the OCM system will be the addition of a fluorescence detection channel to simultaneously acquire coregistered OCM and fluorescence images. The current gold standard to confirm the observation of plaques is to perform histology. In the future, the fluorescence channel could confirm plaque findings. Particularly interesting would be multimodal OCM/fluorescence imaging of curcumin or thioflavin-S-stained brain samples to expand specificity and contrast for the A-β plaques.^64^ Further it would be interesting to investigate tau labeling to explore the possibilities to detect the neurofibrillary tangles. The big advantage of using visible light OCM in combination with fluorescence imaging is that the same light source and illumination path can be used such that the exact same position can be imaged simultaneously.

The presented visible light OCM system has a large variety of potential application fields in neuroimaging. One possibility would be to use the system to investigate brain tumors, to be able to differentiate between healthy and tumorous brain areas. This could be done by using the intensity images and their structural information as shown by Lenz et al.,^65^ by analyzing the attenuation images, as it was shown by Kut et al.^66^ or by the different scattering properties of the tissue as it was shown by Eugui et al.^60^ Through the high axial and transversal resolution of the system, it would be especially interesting to image the microscopic structure of different types of tumors. OCM could also be used to investigate pathological features of other neurological diseases such as multiple sclerosis or Creutzfeldt–Jakob disease. In summary, visible light OCM may be a versatile and powerful tool for neuroimaging.

## 5 Conclusion

*Ex-vivo* brain tissue of patients diagnosed with AD and of a mouse model of AD were investigated using a visible light OCM system. Microscopic anatomical features such as cellular structures, vascular features, and white matter fiber tracts were imaged. Furthermore, amyloid-beta plaques with a diameter in the range of 10 to 70  μm were identified in the cortex. In CAA-affected brain tissue, A-β accumulations were observed in the arterial walls. OCM results were hereby in good agreement with histology. Large fields of view of young and old mice brain sections were imaged using an automated motorized x−y−z stage. A first effort was made to quantify the plaque load using OCM and histology, showing an age-dependent plaque load increase over time. A comparison of the plaque intensity, size, and load in human and mouse cortex tissue was also performed. The analysis showed a significant difference in plaque load and intensity between human and mouse brain tissue. Visible light OCM is a powerful tool to investigate microscopic features in *ex-vivo* human and mouse brain tissue samples and could be extended to many other application fields.
